# A hook wire sliding into pulmonary artery and being extracted under DSA: a case report about a rare complication associated with lung nodule localization

**DOI:** 10.1186/s13019-020-01105-2

**Published:** 2020-04-19

**Authors:** Xu Song, Jie Li, Di Wang

**Affiliations:** 1 Department of Thoracic Surgery, Hwa Mei Hospital, University of Chinese Academy of sciences, Ningbo, China; 2 Ningbo Institute of Life and Health Industry, University of Chinese Academy of Sciences, Ningbo, China; 3 Department of Vascular Surgery, Hwa Mei Hospital, University of Chinese Academy of sciences, Ningbo, China

**Keywords:** Lung nodule, Video-assisted thoracic surgery (VATS), Hook wire, Digital Substraction angiography (DSA)

## Abstract

**Background:**

CT-guided hook wire has been recognized to be a safe and effective percutaneous localizer to identify small pulmonary lesions with ground-glass opacity (GGO) component, while several association complications including pneumothorax, hemothorax, intrapulmonary hemorrhaging, aeroembolism and dislodgement have been reproted. However, sliding into pulmonary artery is an extremly rare comlication of hook wire localization.

**Case presentation:**

A 61-year-old male suffered from multiple pulmonary nodules received right upper lobectomy and right lower lobe wedge resection by video-assisted thoracic surgery (VATS) 3 months ago. Since it might be difficult to identify the ground-glass opacity located in the right lower lobe, a CT-guided hook wire was placed before surgery. During the operation, the hook wire unexpectedly slided into left upper lobe pulmonary artery. With the help of vascular surgery department, the hook wire was extracted by interventional therapy under digital substraction angiography (DSA). The patient was eventually recovered and discharged.

**Conclusions:**

During localization procedure, the tip of hook wire should be far from pulmonary vessels. At the beginning of the operation, the hook wire might as well be removed first. Even if the hook wire was still required to be in the pulmonary parenchyma, it should be fixed to the pleural by a titanic clip or a hemolock clip.

## Background

The growing popularity and efficacy of high-resolution computed tomography (HRCT) has led to an increase in the identification rate of small pulmonary lesions. Although it is possible to identify many small lesions by manual palpation, identification of small lesions with ground-glass opacity (GGO) component or deep lesions away from pleura remains difficult [1]. CT-guided hook wire is currently one of the most used percutaneous localizers [2]. Association complications including pneumothorax, hemothorax, intrapulmonary hemorrhaging, aeroembolism and dislodgement have been reproted [3]. However, sliding into pulmonary artery is an extremly rare comlication of hook wire localization [4].

## Case presentation

**Fig. 1:**
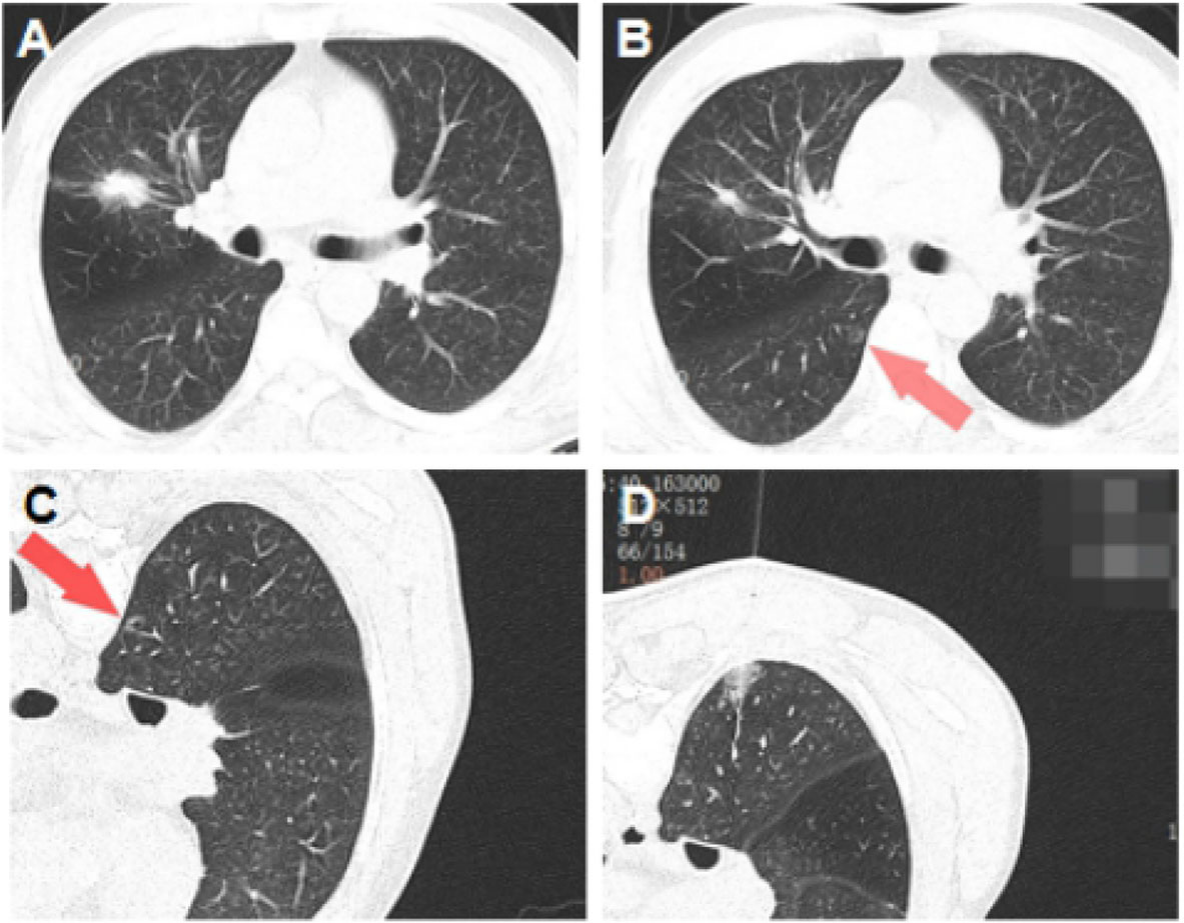
**a** The SPN located at the right upper lobe; **b** The red arrow indicates the GGO that was located at the right lower lobe; **c** The patient received localization procedure in prone position; **d** CT scan after localizer placement, from which we can perceive the tip of the hook wire was too close to a a side branch of basal segmental pulmonary artery. SPN, solid pulmonary nodule; GGO, ground-glass opacity; CT, computed tomography

A 61-year-old gentleman admitted 3 months ago because of multiple pulmonary nodules which had been found in health examination. HRCT showed that there were two nodules, including one solid pulmonary nodule (SPN) that was located at the right upper lobe (Fig. 1a) and one GGO at the right lower lobe (Fig. 1b). Other preoperative examinations showed no contraindications. A right upper lobectomy and right lower lobe wedge resection by single-port video-assisted thoracic surgery (VATS) were performed, and about 3 h before the operation, a CT-guided hook wire localization of the GGO was performed in radiology department. According to the CT scan, the hook wire was inserted 3 cm deep into the pulmonary parenchyma and the hook-shaped tip was fixed 1.8 cm away from the GGO (Fig. 1c, d). There was no pneumothorax or hemothorax during this procedure.

**Fig. 2:**
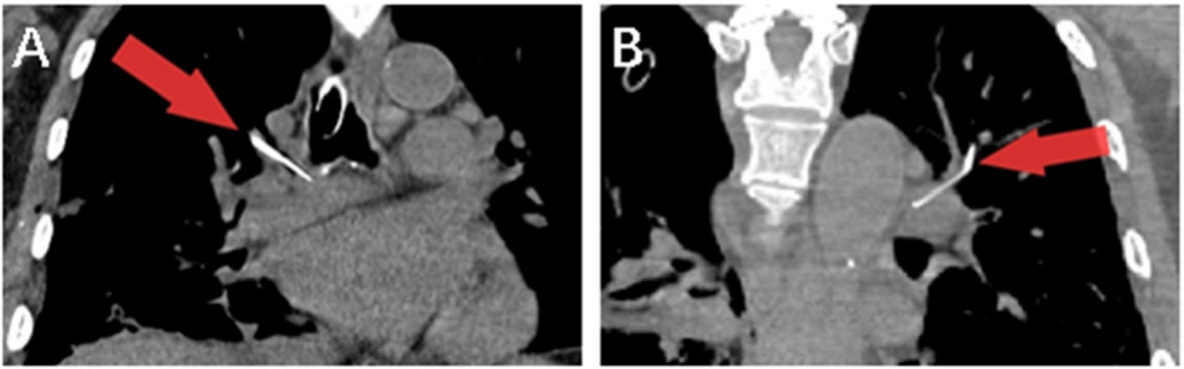
CT scans during the operatin. **a** In the first CT scan, the red arrow indicates the hook wire sliding into artery of apical segment of right upper lobe; **b** In the second CT scan, the red arrow indicates the localizer sliding to the artery of apicoposterior segment of left upper lobe. CT, computed tomography

General anesthesia was performed and then a 4 cm incision was made at the pivot of the fourth intercostal space and the anterior axillary line. While entering the thorax, the hookwire was found still attached to the right lower lobe and the tail outside the thorax was cut off to avoid bacterial contamination. As we planed before, the first step of the operation was wedge resection of the right lower lobe. After several attempts to search for the GGO by manual palpation, the hookwire was submerged in pulmonary parenchyma and could not be touched. A 1 cm incision at the pivot of the eighth intercostal space and the midaxillary line and a 3 cm incision at the pivot of the eighth intercostal space and the posterior axillary line were added but still helpless. Then the patient was sent to the radiologist department where a CT scan was performed and the hookwire was found in the artery of apical segment of right upper lobe, which was planned to be removed (Fig. 2a). When the patient was sent back to the theater, the operation continued from right lower lobe wedge resection, then right upper lobectomy. After successfully dissecting pulmonary vein and superior bronchus of the right upper lobe with endoscopic stapler (Ethicon), we ligatured and cut arteries of right upper lobe one by one manually but did not find the hookwire. Afterwards, a second CT scan was performed and the hookwire was found in the artery of apicoposterior segment of left upper lobe (Fig. 2b). Since this foreign body could not be reached through right thoracic cavity, the vascular surgery department was consulted after the incisions were closed. Following the suggestion of consultation, the patient was prepared to receive interventional therapy under digital substraction angiography (DSA).

**Fig. 3:**
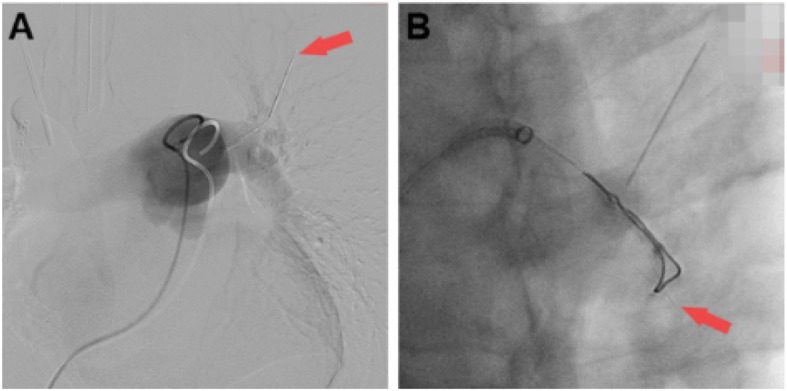
Interventional therapy under DSA. **a** The red arrow indicates the hook wire; **b** The red arrow indicates the hook-shaped tip was catched by the endoloop. DSA, digital substraction angiography

As shown on the DSA images, the hook wire was still in the artery of apicoposterior segment of left upper lobe (Fig. 3a). A guide wire was inserted through right femoral vein to the target artery, subsequently, an endoloop was inserted to hitch the hookwire and extract it out (Fig. 3b). During the process of DSA, there was no bradycardia, tachycardia or arrhythmia.

The patient was eventually discharged 15 days after surgery. Low molecular weight heparin was injected in subcutaneous tissue two times a day from the second day after surgery to discharge day. The pathological results indicated that the SPN in right upper lobe was an invasive adenocarcinoma, while the GGO in right lower lobe was a minimally invasive adenocarcinoma, and there was no metastatic lymph node. The patient received follow-up CT scan 3 months after surgery, which showed no obvious abnormality.

## Discussion and conclusions

Small pulmonary nodules, especially the GGOs, are often difficult to identify during thoracoscopic resection, and preoperative CT-guided localization can be helpful [5]. Hook wire, which is recognized to be safe and effective, is currently one of the most used percutaneous localizers [2]. However, several associated complications including pneumothorax, hemothorax, intrapulmonary hemorrhaging, aeroembolism have been reported and have a low incidence [3]. Nevertheless, a hook wire sliding into pulmonary is an extremly rare complication, and being extracted by interventional therapy under DSA, as a remedial measure, has never been reported yet accroding to medical literature retrieval.

In this case, we speculate that the tip of hook wire was too close to or even inserted into a side branch of basal segmental pulmonary artery in localization procedure. During the operation, the hook wire was pressed into the artery by repeatedly manual palpation, then it wandered back and forth through right pulmonary artery, right ventricle and left pulmonary artery benifit from the smooth lining of blood vessels. Fortunately, since the hook wire slided into the artery of apicoposterior segment of left upper lobe, the hook-shaped tip pointing to proximal end was more easily to be hitched by an endoloop under DSA.

This case provides us with several valuable lessons. First, the needle tip should be far from pulmonary vessels during the localization procedure. Second, the hook wire might as well be removed at the beginning of the operation since the pleural wound caused by the hook-shaped tip can be easily recognized. If the hook wire was still required, it should be fixed to the pleural by a titanic clip or a hemolock clip.

## Data Availability

The medical records of this patient are available in patient record system of Hwa Mei hospital, University of Chinese academy of sciences.

## Abbreviations

DSA: Digital substraction angiography
GGO: Ground-glass opacity
HRCT: High-resolution computed tomography
SPN: Solid pulmonary nodule
VATS: Video-assisted thoracic surgery
